# Supplementary material for: Extending Protein Language Models to a Viral Genomic Scale Using Biologically Induced Sparse Attention
Source: bioRxiv. 2025 Jun 1:2025.05.29.656907. Preprint. [Version 1] doi: 10.1101/2025.05.29.656907 (PMC12154925; doi:10.1101/2025.05.29.656907)
Supplement: Supplementary file 1 [file NIHPP2025.05.29.656907V1-supplement-1.pdf]

bioRxiv preprint doi: https://doi.org/10.1101/2025.05.29.656907; this version posted June 1, 2025. The copyright holder for this preprint (which was not certified by peer review) is the author/funder, who has granted bioRxiv a license to display the preprint in perpetuity. It is made available under aCC-BY 4.0 International license.

Supplementary Material

Using embeddings to predict taxonomy

To assess the taxonomic discriminability of the learned embeddings, we conducted a supervised classification analysis. For each protein or genome in the dataset, a 1,280-dimensional embedding vector was generated and paired with its corresponding taxonomic label. The dataset, comprising 100,000 proteins and 5,000 genomes, was randomly partitioned into training (80%) and validation (20%) subsets. To ensure a robust evaluation, we restricted the analysis to the top X most frequently represented taxonomic species. A single-layer neural network with an intermediate hidden dimension of 2,560 was trained for 1,000 epochs using a learning rate of 1e-3. Model performance was evaluated using 5-fold cross-validation, and standard classification metrics were reported.

NCBI Virus Database

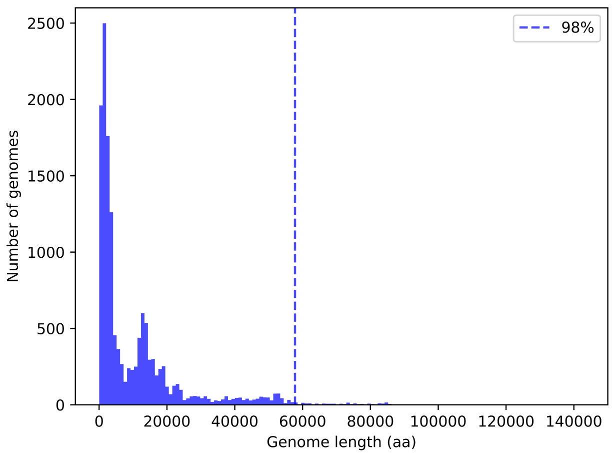

Figure 13: **Viral genome length distributions in the NCBI Virus Database.** This histogram shows that a context length of 61k amino acid allows us to process over 98% of the 14,436 complete geomes of the database.

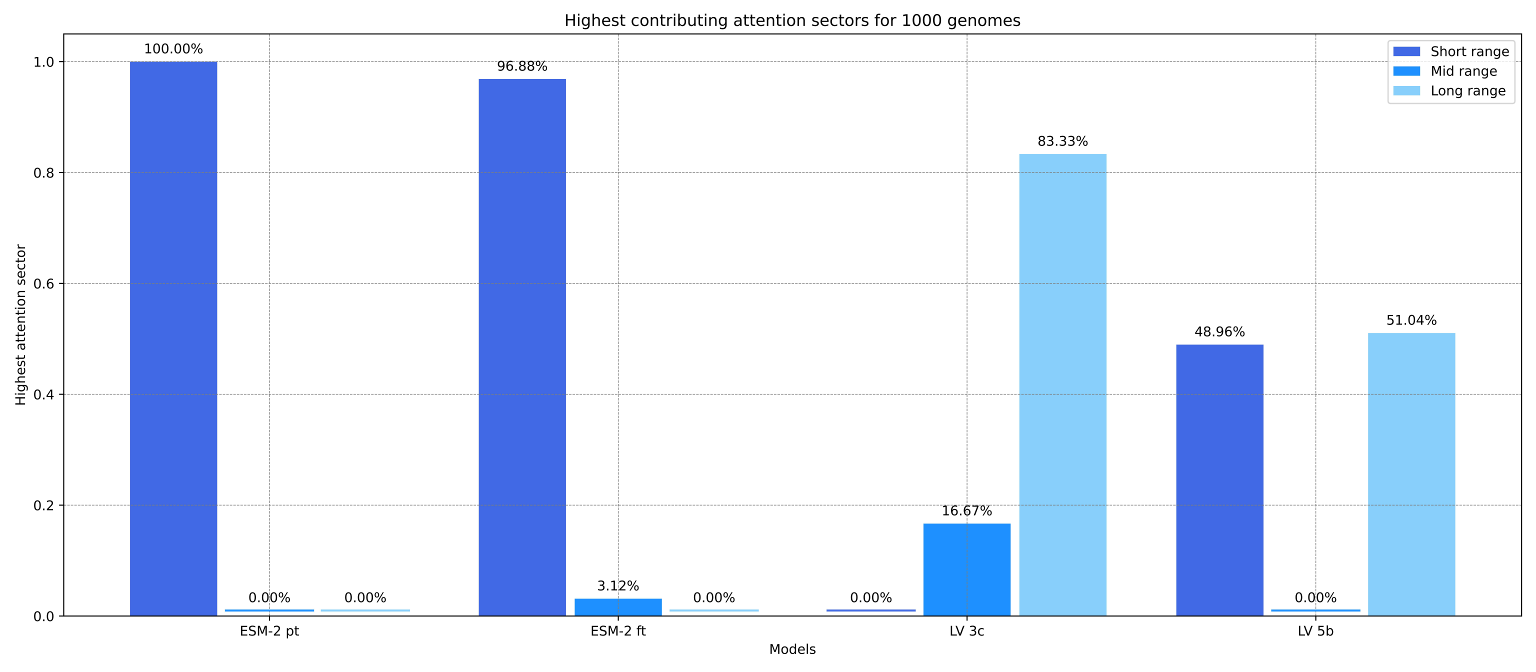
Highest contributing attention sectors for 1000 genomes
Short range
Mid range
Long range
100.00%
0.00%
0.00%
96.88%
3.12%
0.00%
0.00%
16.67%
83.33%
48.96%
0.00%
51.04%
1.0
0.8
0.6
0.4
0.2
0.0
Highest attention sector
ESM-2 pt
ESM-2 ft
LV 3c
LV 5b
Models

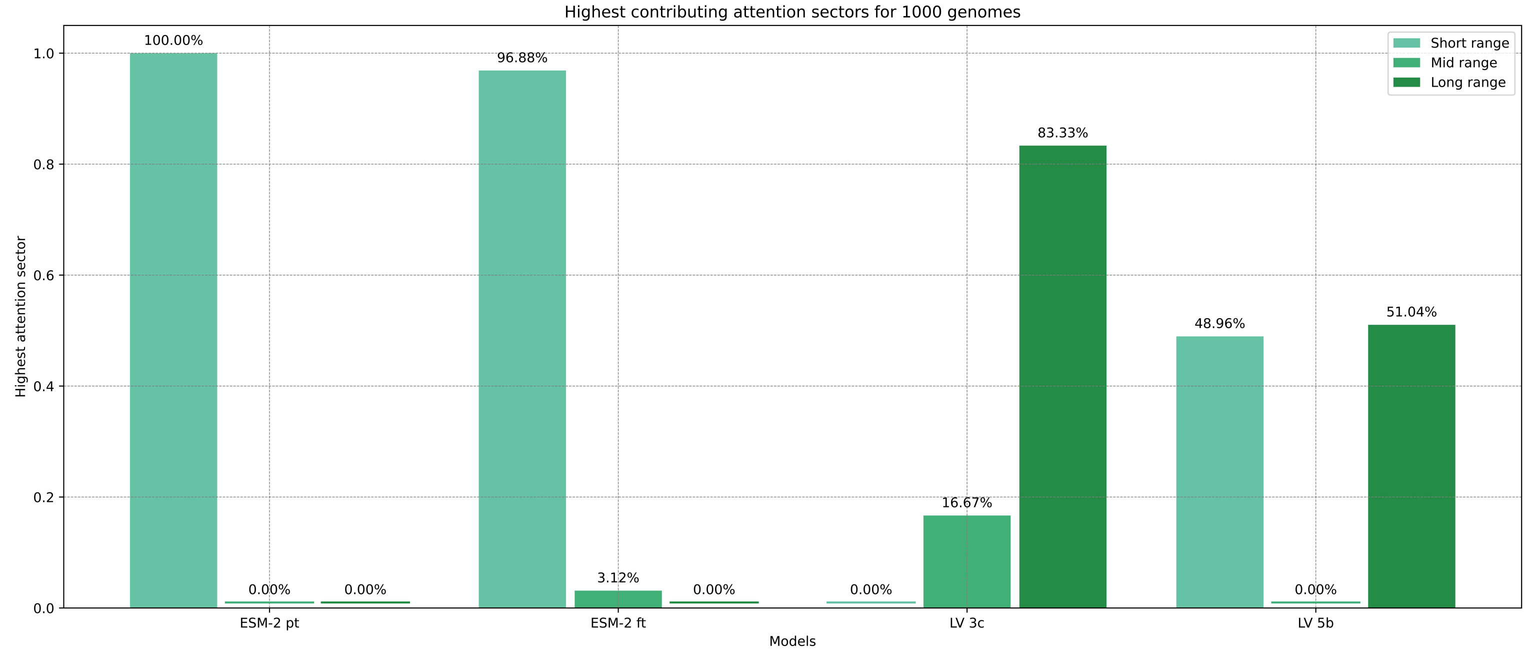
Highest contributing attention sectors for 1000 genomes
Short range
Mid range
Long range
100.00%
0.00%
0.00%
96.88%
3.12%
0.00%
0.00%
16.67%
83.33%
48.96%
0.00%
51.04%
Highest attention sector
1.0
0.8
0.6
0.4
0.2
0.0
ESM-2 pt
ESM-2 ft
LV 3c
LV 5b
Models

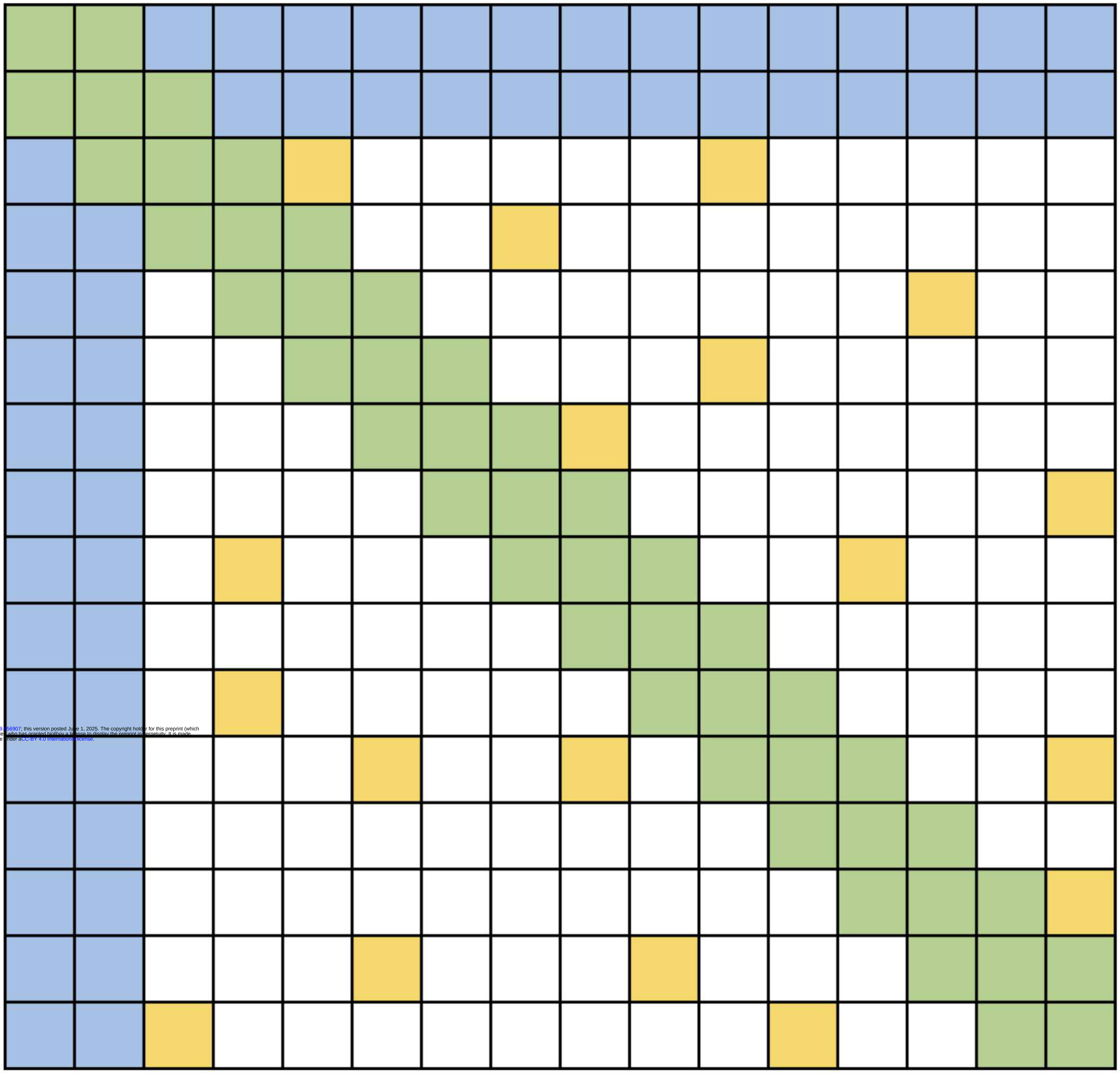

bioRxiv preprint doi: https://doi.org/10.1101/2025.05.29.656907; this version posted June 1, 2025. The copyright holder for this preprint (which was not certified by peer review) is the author/funder, who has granted bioRxiv a license to display the preprint in perpetuity. It is made available under aCC-BY 4.0 International license.

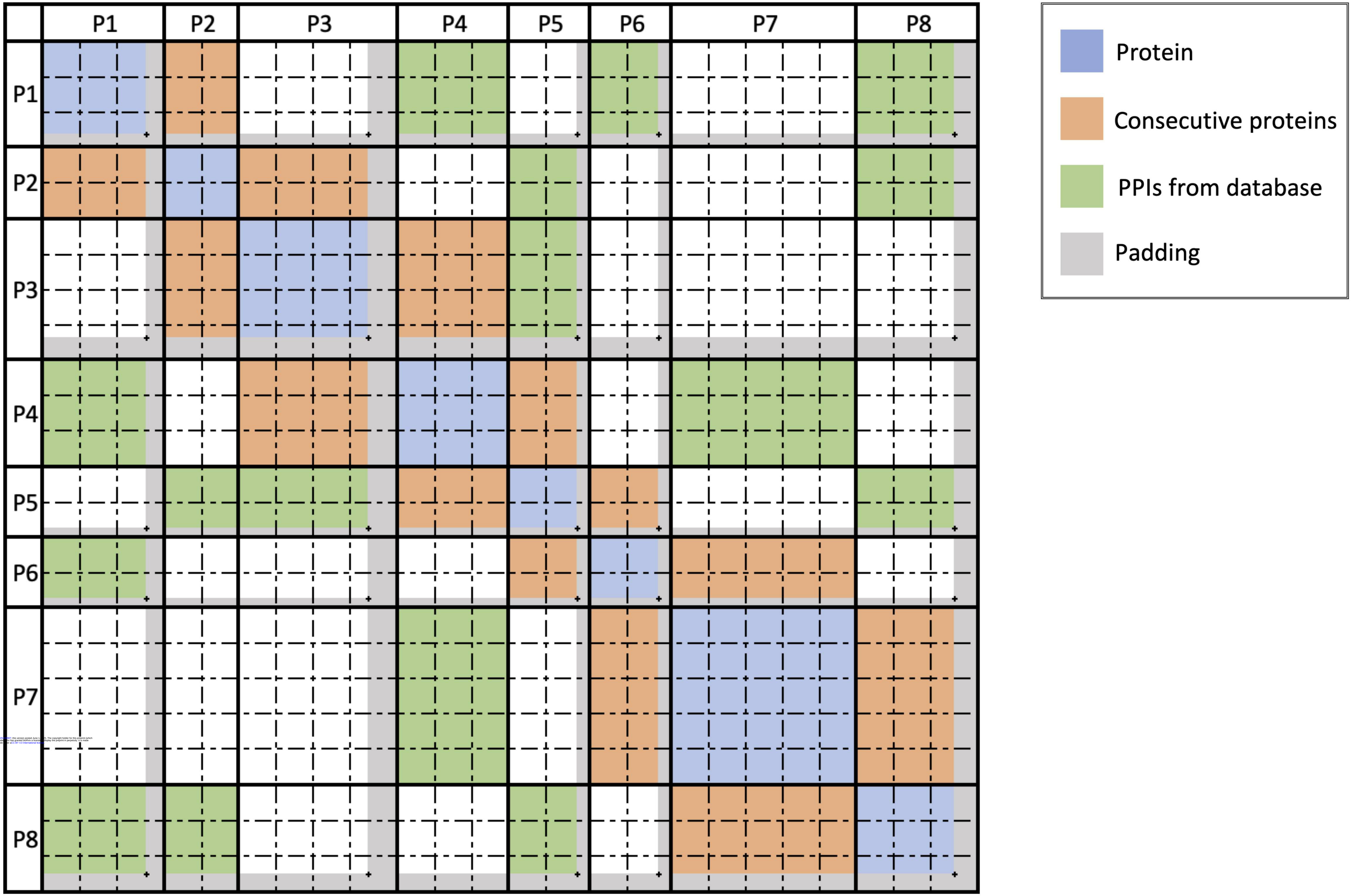

bioRxiv preprint doi: https://doi.org/10.1101/2025.05.29.656907; this version posted June 1, 2025. The copyright holder for this preprint (which was not certified by peer review) is the author/funder, who has granted bioRxiv a license to display the preprint in perpetuity. It is made available under aCC-BY 4.0 International license.

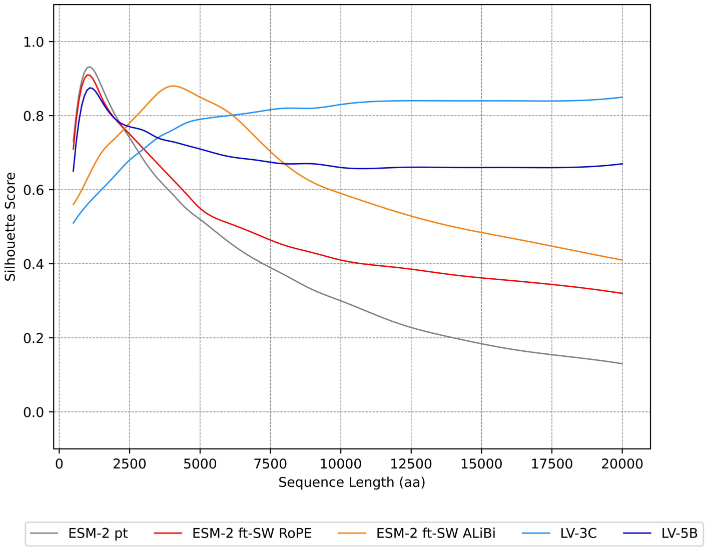

Silhouette Score
Sequence Length (aa)
0.0
0.2
0.4
0.6
0.8
1.0
0
2500
5000
7500
10000
12500
15000
17500
20000
ESM-2 pt
ESM-2 ft-SW RoPE
ESM-2 ft-SW ALiBi
LV-3C
LV-5B

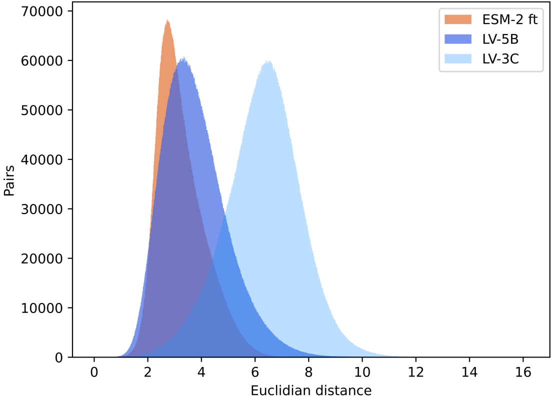

ESM-2 ft
LV-5B
LV-3C
70000
60000
50000
40000
30000
20000
10000
0
Pairs
0
2
4
6
8
10
12
14
16
Euclidian distance

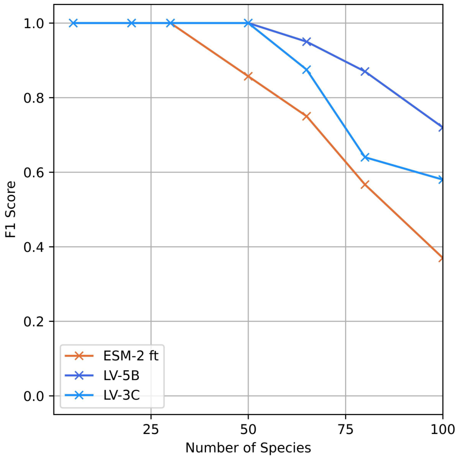

1.0
0.8
0.6
0.4
0.2
0.0
F1 Score
25
50
75
100
Number of Species
ESM-2 ft
LV-5B
LV-3C

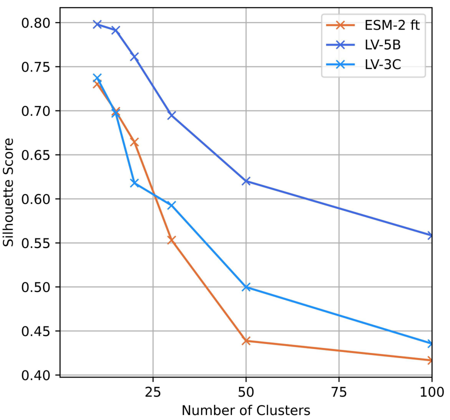

ESM-2 ft
LV-5B
LV-3C
Silhouette Score
Number of Clusters
0.80
0.75
0.70
0.65
0.60
0.55
0.50
0.45
0.40
25
50
75
100

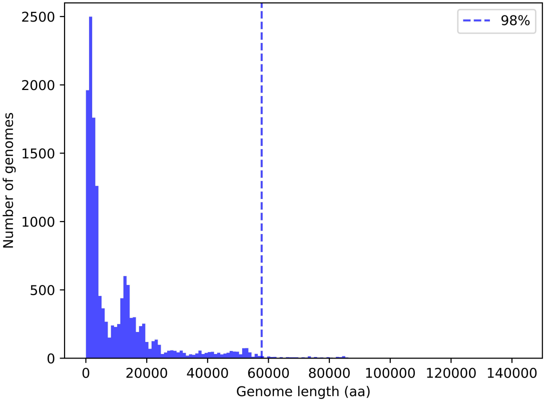

98%
Number of genomes
2500
2000
1500
1000
500
0
0
20000
40000
60000
80000
100000
120000
140000
Genome length (aa)

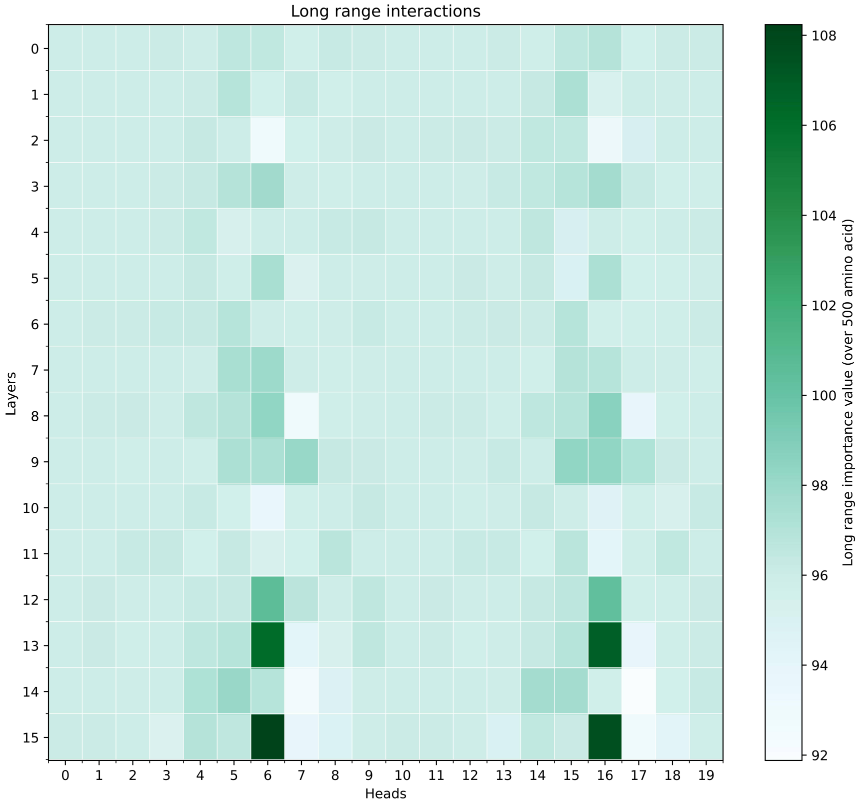
Long range interactions
Layers
0
1
2
3
4
5
6
7
8
9
10
11
12
13
14
15
Heads
0
1
2
3
4
5
6
7
8
9
10
11
12
13
14
15
16
17
18
19
Long range importance value (over 500 amino acid)
108
106
104
102
100
98
96
94
92

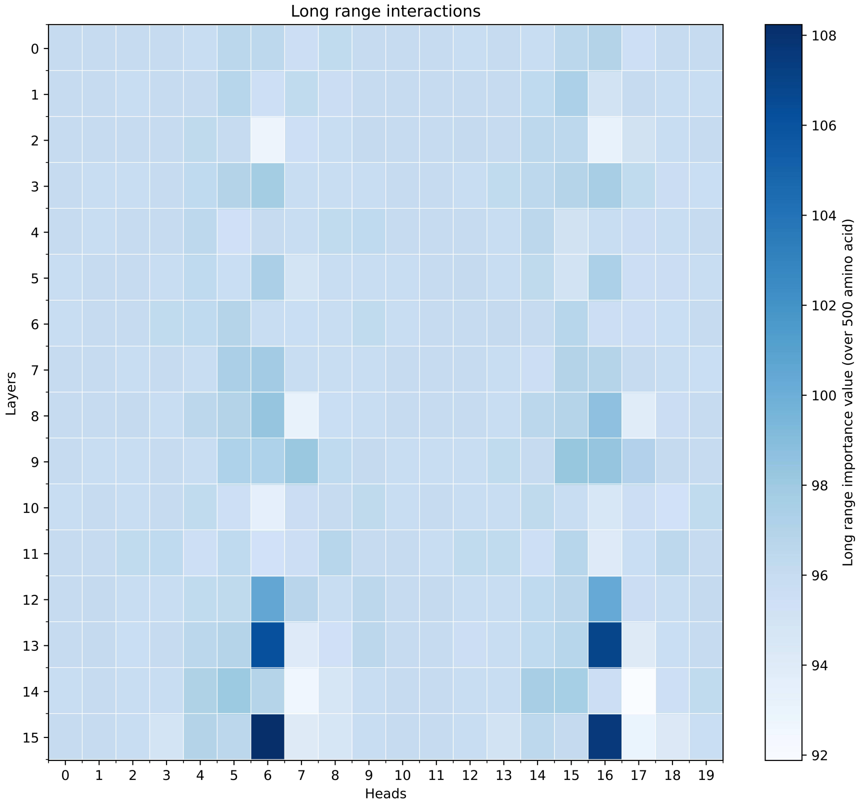

Long range interactions
Layers
Heads
0
1
2
3
4
5
6
7
8
9
10
11
12
13
14
15
16
17
18
19
Long range importance value (over 500 amino acid)
108
106
104
102
100
98
96
94
92

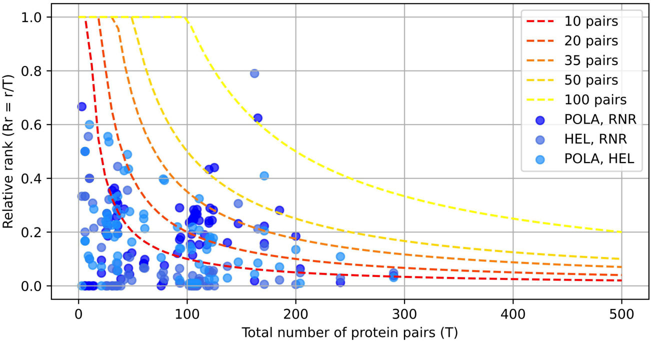
10 pairs
20 pairs
35 pairs
50 pairs
100 pairs
POLA, RNR
HEL, RNR
POLA, HEL
Relative rank (Rr = r/T)
Total number of protein pairs (T)
0.0
0.2
0.4
0.6
0.8
1.0
0
100
200
300
400
500

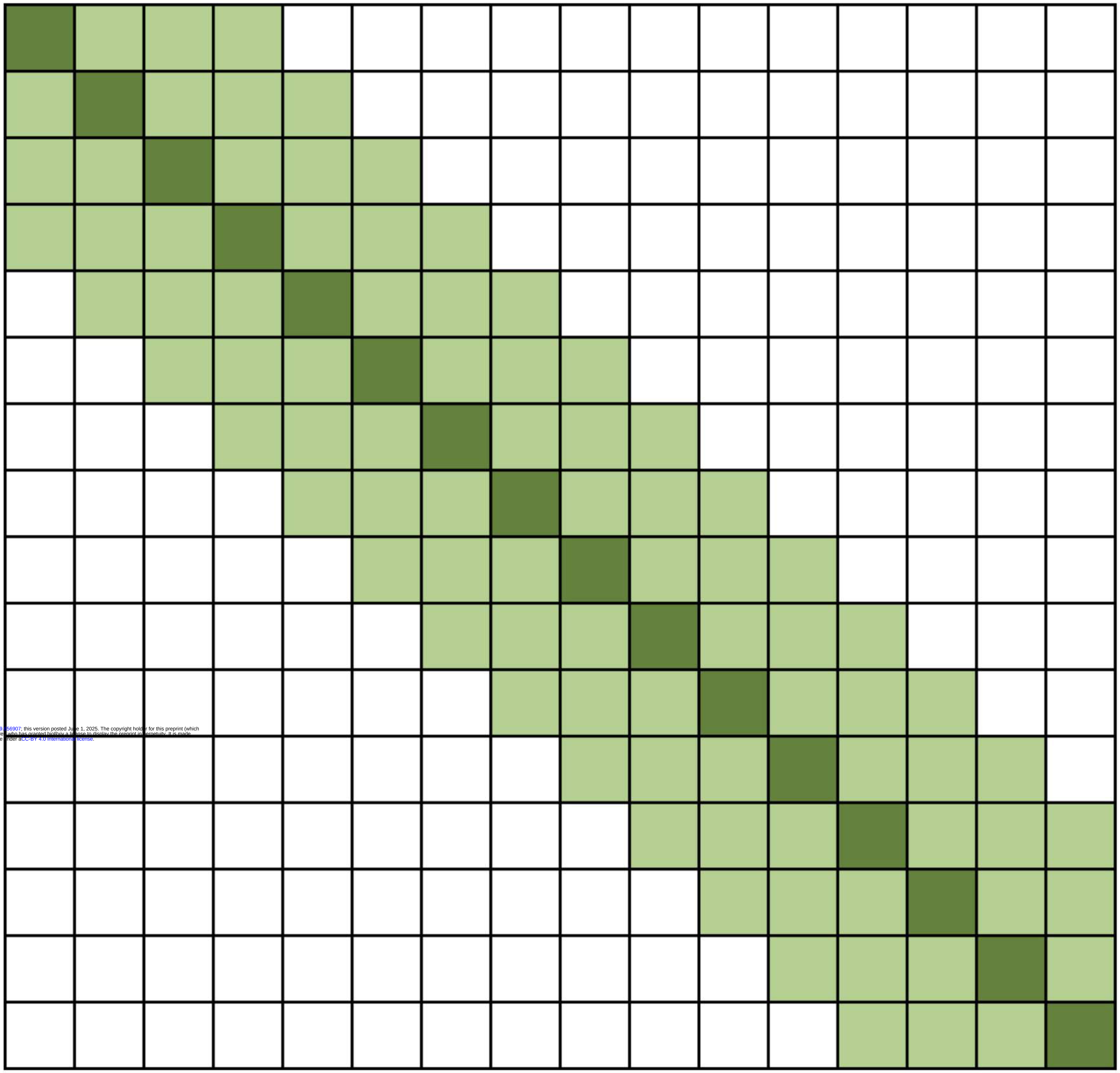

bioRxiv preprint doi: https://doi.org/10.1101/2025.05.29.656907; this version posted June 1, 2025. The copyright holder for this preprint (which was not certified by peer review) is the author/funder, who has granted bioRxiv a license to display the preprint in perpetuity. It is made available under aCC-BY 4.0 International license.

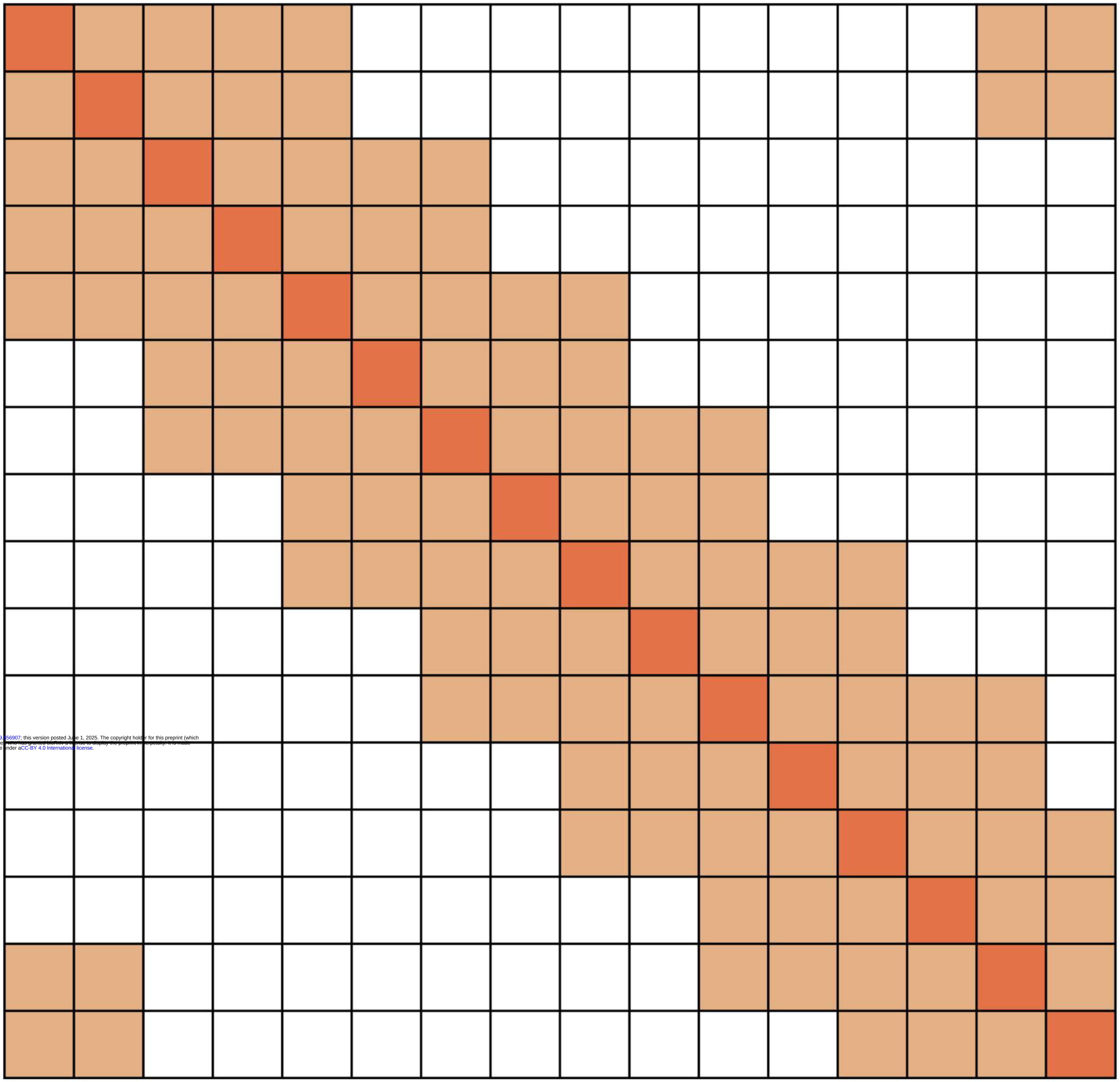

bioRxiv preprint doi: https://doi.org/10.1101/2025.05.29.656907; this version posted June 1, 2025. The copyright holder for this preprint (which was not certified by peer review) is the author/funder, who has granted bioRxiv a license to display the preprint in perpetuity. It is made available under aCC-BY 4.0 International license.

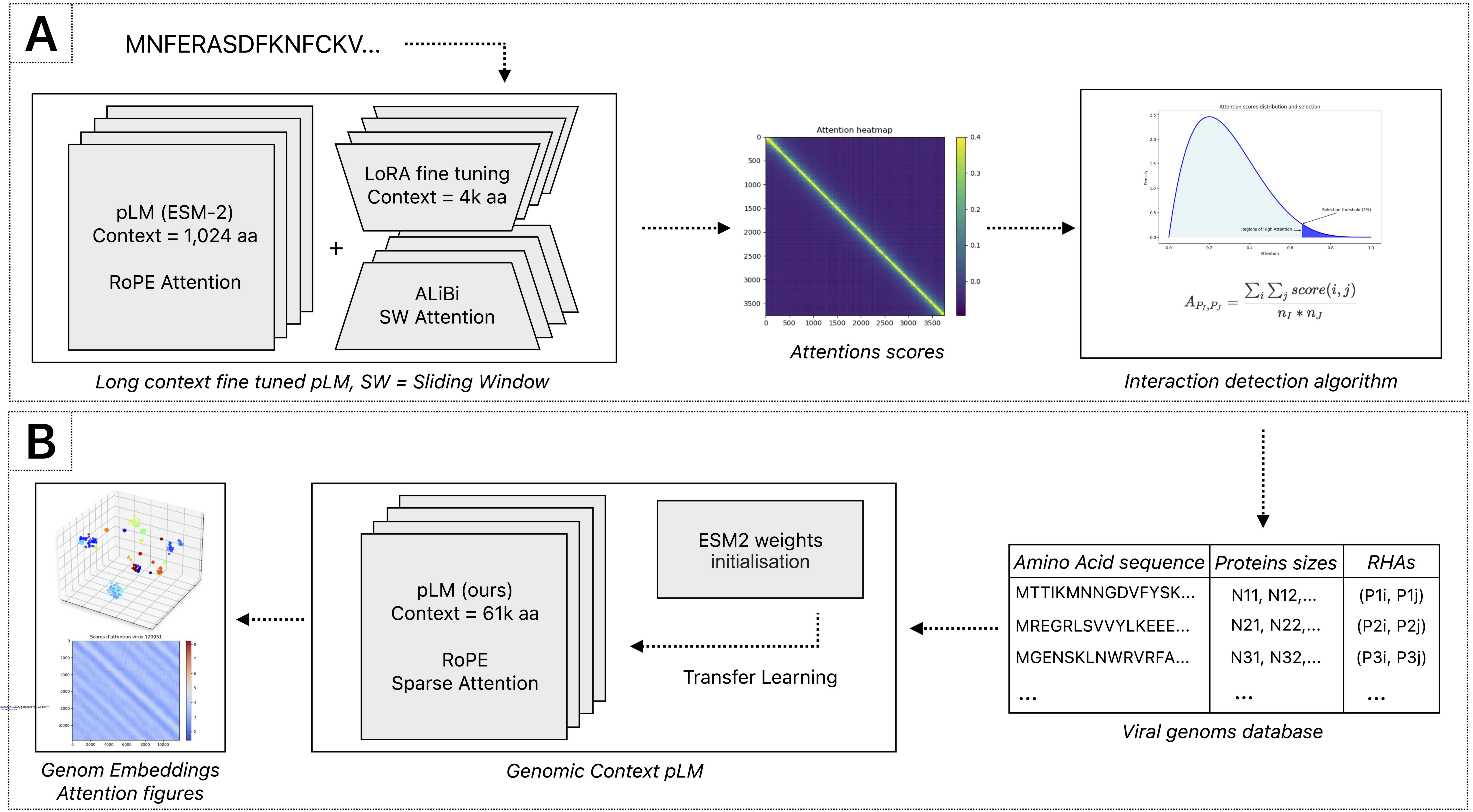

A
MNFERASDFKNFCKV...
pLM (ESM-2)
Context = 1,024 aa
RoPE Attention
+
LoRA fine tuning
Context = 4k aa
ALiBi
SW Attention
Long context fine tuned pLM, SW = Sliding Window
Attention heatmap
Attentions scores
Attention scores distribution and selection
Density
Attention
Selection threshold (2%)
Regions of High Attention
$A_{P_I,P_J} = \frac{\sum_i \sum_j score(i,j)}{n_I * n_J}$
Interaction detection algorithm
B
Scores d'attention virus 129951
Genom Embeddings
Attention figures
pLM (ours)
Context = 61k aa
RoPE
Sparse Attention
ESM2 weights
initialisation
Transfer Learning
Genomic Context pLM
Amino Acid sequence
Proteins sizes
RHAs
MTTIKMNNGDVFYSK...
N11, N12,...
(P1i, P1j)
MREGRLSVVYLKEEE...
N21, N22,...
(P2i, P2j)
MGENSKLNWRVRFA...
N31, N32,...
(P3i, P3j)
...
Viral genoms database

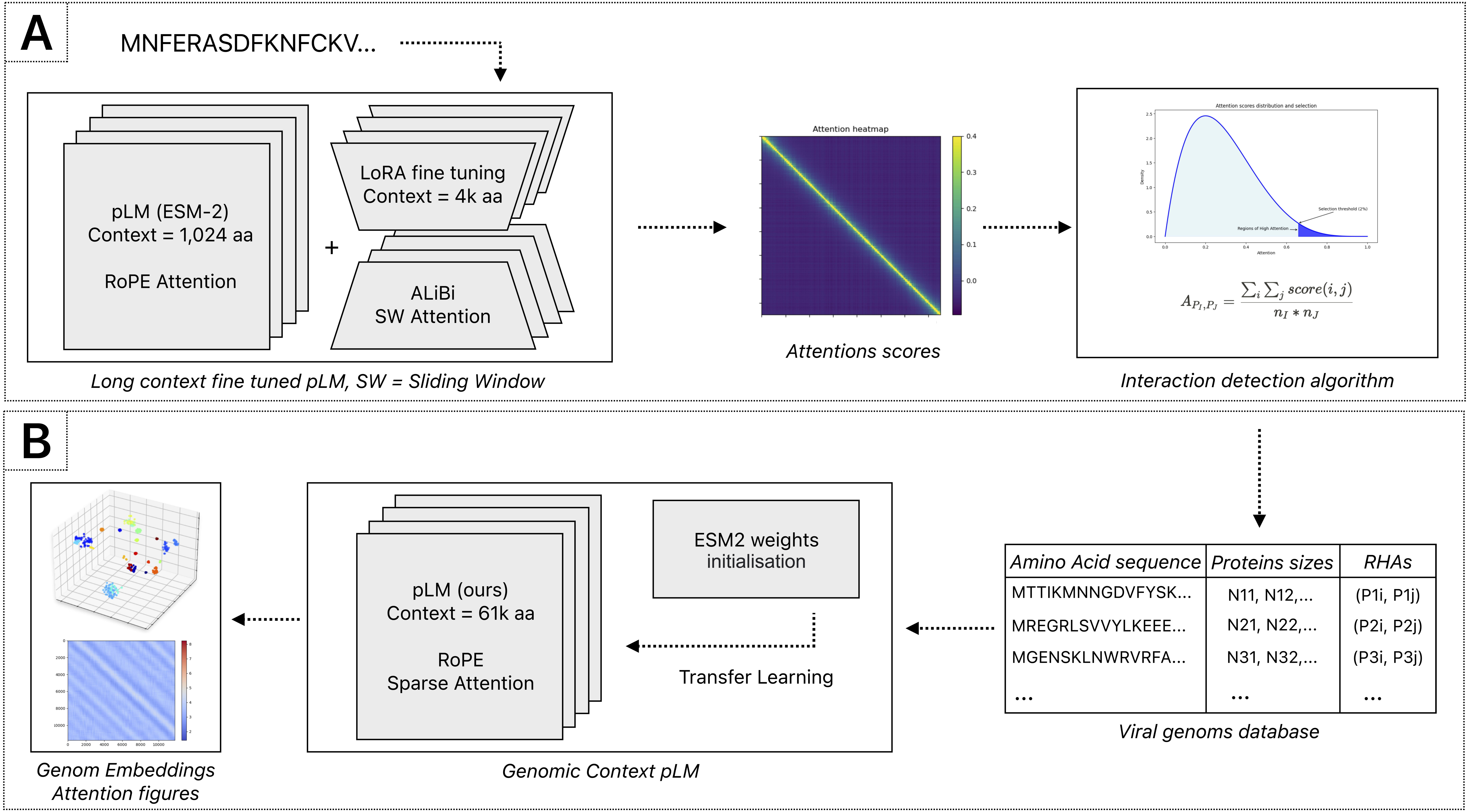
A
MNFERASDFKNFCKV...
pLM (ESM-2)
Context = 1,024 aa
RoPE Attention
+
LoRA fine tuning
Context = 4k aa
ALiBi
SW Attention
Long context fine tuned pLM, SW = Sliding Window
Attention heatmap
Attentions scores
Attention scores distribution and selection
Selection threshold (2%)
Regions of High Attention
Density
Attention
$A_{P_I,P_J} = \frac{\sum_i \sum_j score(i,j)}{n_I * n_J}$
Interaction detection algorithm
B
Genom Embeddings
Attention figures
pLM (ours)
Context = 61k aa
RoPE
Sparse Attention
ESM2 weights
initialisation
Transfer Learning
Genomic Context pLM
Amino Acid sequence
Proteins sizes
RHAs
MTTIKMNNGDVFYSK...
N11, N12,...
(P1i, P1j)
MREGRLSVVYLKEEE...
N21, N22,...
(P2i, P2j)
MGENSKLNWRVRFA...
N31, N32,...
(P3i, P3j)
...
...
...
Viral genoms database

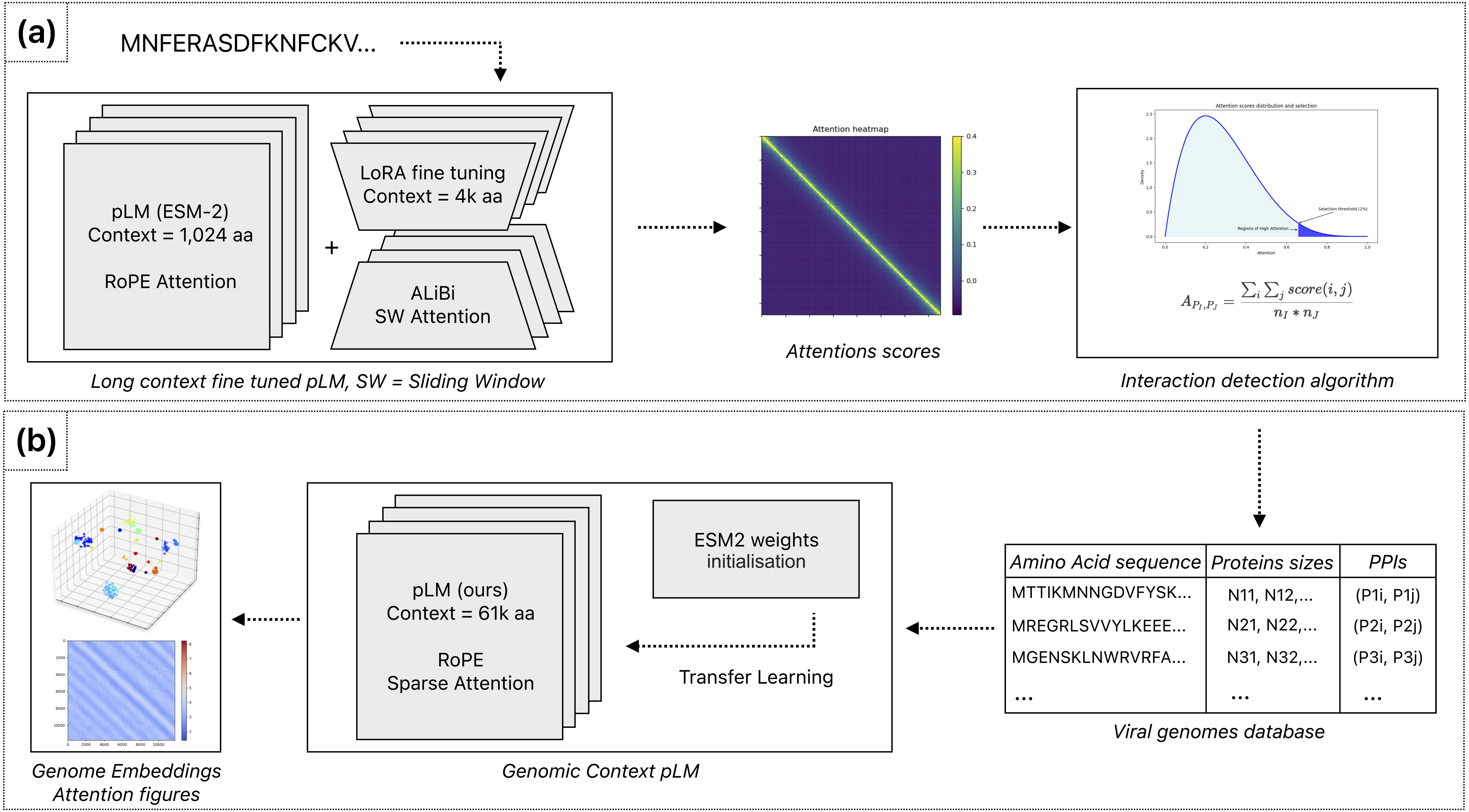
(a)
MNFERASDFKNFCKV...
pLM (ESM-2)
Context = 1,024 aa
RoPE Attention
+
LoRA fine tuning
Context = 4k aa
ALiBi
SW Attention
Long context fine tuned pLM, SW = Sliding Window
Attention heatmap
Attentions scores
Attention scores distribution and selection
Selection threshold (2%)
Regions of High Attention
Density
Attention
$A_{P_I,P_J} = \frac{\sum_i \sum_j score(i,j)}{n_I * n_J}$
Interaction detection algorithm
(b)
Genome Embeddings
Attention figures
pLM (ours)
Context = 61k aa
RoPE
Sparse Attention
ESM2 weights
initialisation
Transfer Learning
Genomic Context pLM
Amino Acid sequence
Proteins sizes
PPIs
MTTIKMNNGDVFYSK...
N11, N12,...
(P1i, P1j)
MREGRLSVVYLKEEE...
N21, N22,...
(P2i, P2j)
MGENSKLNWRVRFA...
N31, N32,...
(P3i, P3j)
...
Viral genomes database

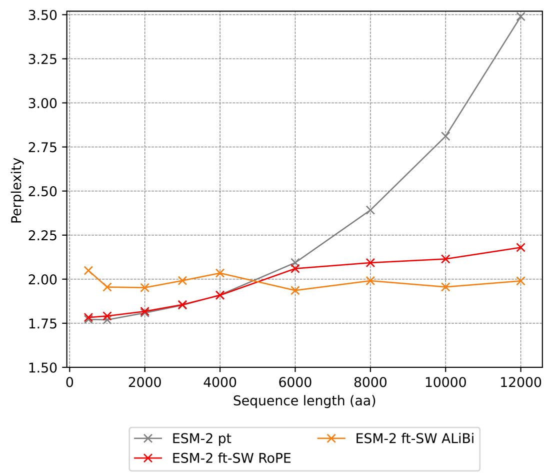
Perplexity
Sequence length (aa)
3.50
3.25
3.00
2.75
2.50
2.25
2.00
1.75
1.50
0
2000
4000
6000
8000
10000
12000
ESM-2 pt
ESM-2 ft-SW RoPE
ESM-2 ft-SW ALiBi

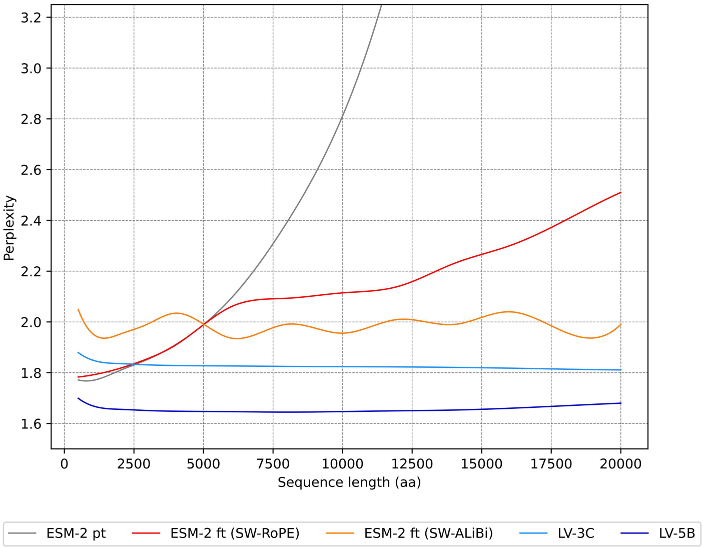
Perplexity
3.2
3.0
2.8
2.6
2.4
2.2
2.0
1.8
1.6
0
2500
5000
7500
10000
12500
15000
17500
20000
Sequence length (aa)
ESM-2 pt
ESM-2 ft (SW-RoPE)
ESM-2 ft (SW-ALiBi)
LV-3C
LV-5B

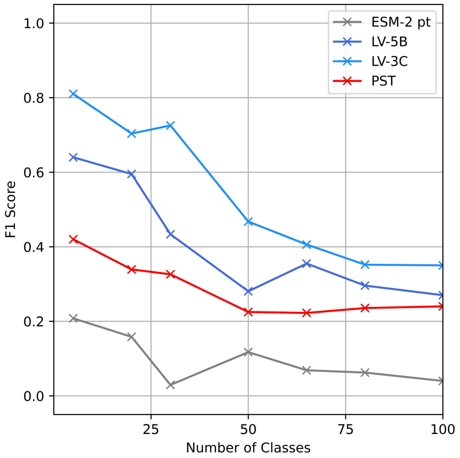

ESM-2 pt
LV-5B
LV-3C
PST
F1 Score
0.0
0.2
0.4
0.6
0.8
1.0
25
50
75
100
Number of Classes

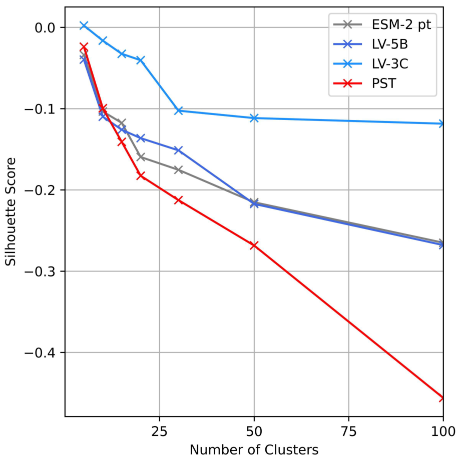
ESM-2 pt
LV-5B
LV-3C
PST
Silhouette Score
0.0
−0.1
−0.2
−0.3
−0.4
25
50
75
100
Number of Clusters

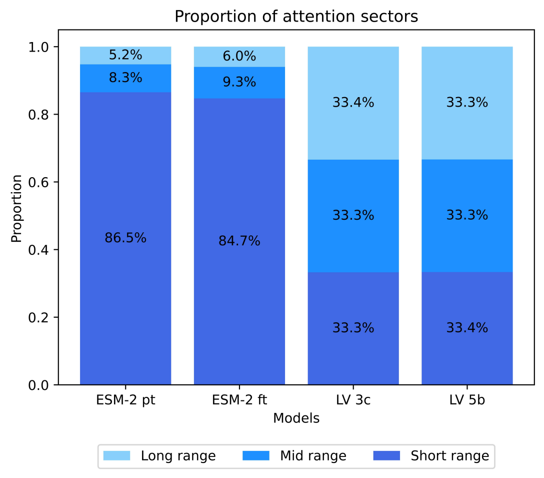

Proportion of attention sectors
1.0
0.8
0.6
0.4
0.2
0.0
Proportion
5.2%
8.3%
86.5%
6.0%
9.3%
84.7%
33.4%
33.3%
33.3%
33.3%
33.3%
33.4%
ESM-2 pt
ESM-2 ft
LV 3c
LV 5b
Models
Long range
Mid range
Short range

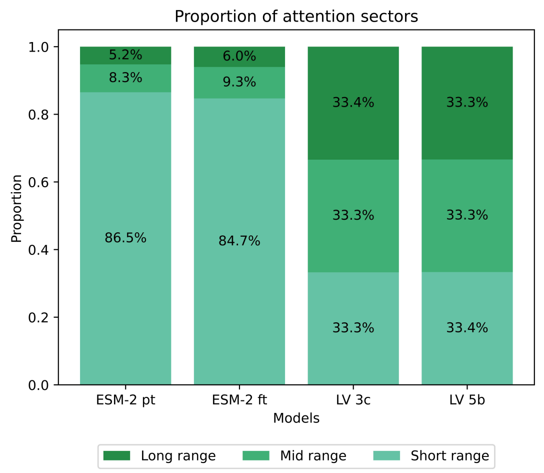
Proportion of attention sectors
Proportion
Models
ESM-2 pt
ESM-2 ft
LV 3c
LV 5b
5.2%
8.3%
86.5%
6.0%
9.3%
84.7%
33.4%
33.3%
33.3%
33.3%
33.3%
33.4%
Long range
Mid range
Short range
0.0
0.2
0.4
0.6
0.8
1.0

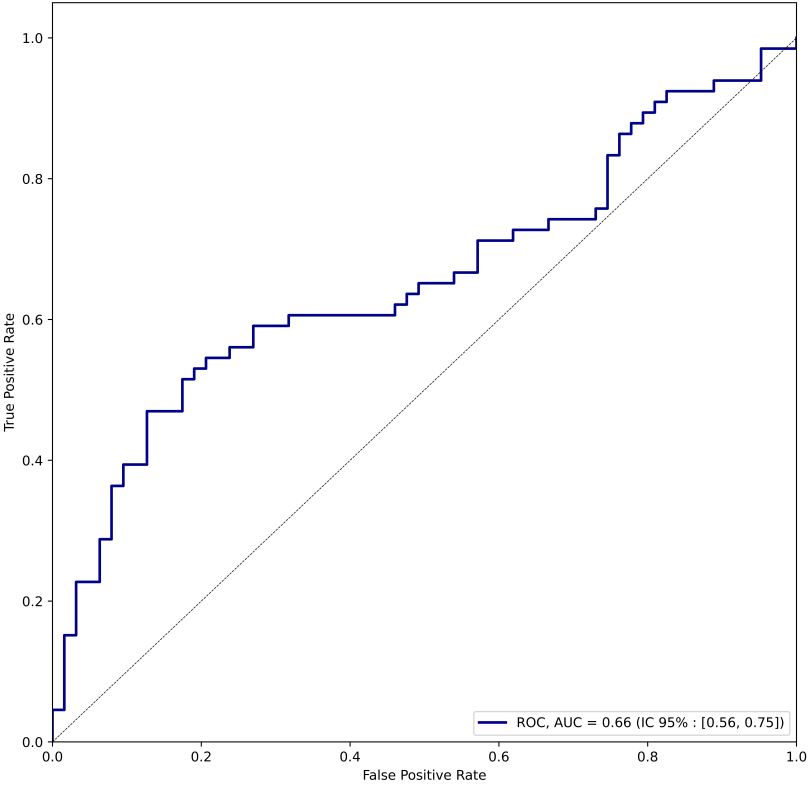
True Positive Rate
False Positive Rate
0.0
0.2
0.4
0.6
0.8
1.0
ROC, AUC = 0.66 (IC 95% : [0.56, 0.75])

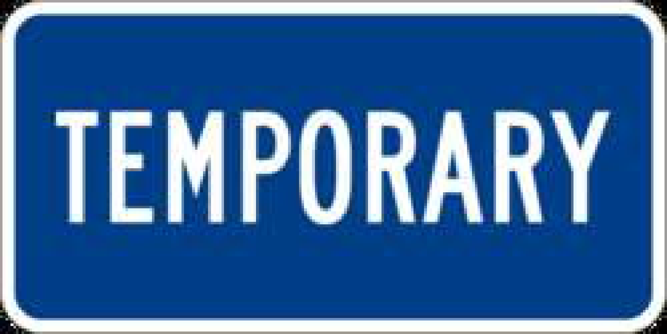
TEMPORARY

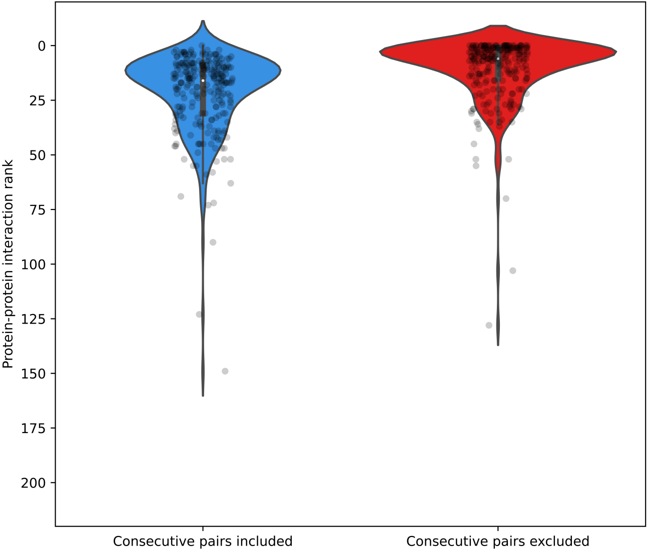

Protein-protein interaction rank
0
25
50
75
100
125
150
175
200
Consecutive pairs included
Consecutive pairs excluded
